# Environmental tobacco smoke exposure exaggerates bleomycin-induced collagen overexpression during pulmonary fibrogenesis

**DOI:** 10.1186/s12950-024-00377-y

**Published:** 2024-03-20

**Authors:** Qixin Wang, Chiara Goracci, Isaac Kirubakaran Sundar, Irfan Rahman

**Affiliations:** 1 Department of Environmental Medicine, University of Rochester Medical Center, 601 Elmwood Avenue, Box 850, Rochester, NY 14642 USA; 2 Department of Internal Medicine, Division of Pulmonary, Critical Care and Sleep Medicine, University of Kansas Medical Center, Kansas City, KS USA

**Keywords:** Environmental tobacco smoke, Fibrosis, Collagen, C3 system, Aging

## Abstract

Environmental tobacco smoke (ETS) is known to cause lung inflammatory and injurious responses. Smoke exposure is associated with the pathobiology related to lung fibrosis, whereas the mechanism that ETS exposure augments pulmonary fibrogenesis is unclear. We hypothesized that ETS exposure could exacerbate fibrotic responses via collagen dynamic dysregulation and complement activation. C57BL/6J and p16-3MR mice were exposed to ETS followed by bleomycin administration. ETS exposure exacerbated bleomycin-induced collagen and lysyl oxidase overexpression in the fibrotic lesion. ETS exposure also led to augmented bleomycin-induced upregulation of C3 and C3AR, which are pro-fibrotic markers. Moreover, overexpressed collagens and C3 levels were highly significant in males than females. The old mice (17 months old) were exposed to ETS and treated with bleomycin to induce fibrogenesis which is considered as an aging-associated disease. Fewer gene and protein dysregulations trends were identified between ETS exposure with the bleomycin group and the bleomycin alone group in old mice. Based on our findings, we suggested that ETS exposure increases the risk of developing severe lung fibrotic responses via collagen overexpression and lysyl oxidase-mediated collagen stabilization in the fibrotic lesion, and potentially affected the complement system activation induced by bleomycin. Further, male mice were more susceptible than females during fibrogenesis exacerbation. Thus ETS and bleomycin induced lung fibrotic changes via collagen-lysyl oxidase in an age-dependent mechanism.

**Supplementary Information:**

The online version contains supplementary material available at 10.1186/s12950-024-00377-y.

## Introduction

Tobacco smoke is not only a global public health concern [1], but also one of the major risk factors involved in chronic lung diseases such as chronic obstructive pulmonary disease (COPD) and idiopathic pulmonary fibrosis (IPF) [2, 3]. IPF is a chronic interstitial lung disease with unknown causes [4], and lung fibrosis is usually considered a chronic inflammatory disease with excessive burdens of permanent scar formation in the lungs [5]. The initial stage of fibrogenesis starts with an inflammatory response and activated wound healing process, which contributes to temporary extracellular matrix (ECM) deposition. However, chronic inflammation results in dysregulated repair and continuously accumulated ECM, which could turn into permanent scars.

Tobacco smoke exposure can be considered active smoking exposure or passive smoking exposure. ETS is a mixture of side-stream cigarette smoke (CS) and main-stream CS exhaled by active smokers. It is well known that both CS and ETS could induce a significant inflammatory response, abnormal respiratory syndromes, and CS exposure aggravates fibrotic progression [6–9]. CS exposure exacerbates the collagen deposition and α-smooth muscle actin (αSMA) expression induced by influenza virus (IAV) infection in primary pulmonary fibroblast [7]. Pre-expose to CS also contributes to augmented collagen deposition induced by bleomycin in mouse lungs [6]. One possible mechanism is the TGFβ/Smad2 pathway, which was activated by CS extract (CSE) treatment in lung fibroblasts and pleural mesothelial cells [6]. Except for the animal model, several studies have shown that ex-smoker with IPF exhibit increased and more severe inflammatory responses in the alveolar space [10]. ETS exposure showed increased risks of asthma and COPD development [11, 12]. Our previous study showed that CS exposure could activate epithelial-to-mesenchymal transition and induce collagen deposition in the lungs [13], while no study investigated the role of ETS in exacerbating fibrotic progression. More importantly, lung fibrosis is usually considered as a senescence/aging-associated disease and CS exposure is also well-known to induce premature senescence and aging [14, 15].

ECM is formed with extracellular proteins as basal material to support cells in the three-dimension microenvironment. The ECM dynamics are involved in multiple disease mechanisms and overexpression of ECM is commonly observed in fibrotic lesion. The major ECM proteins are collagens, fibronectin, and elastin. Among collagens, types 1 and 3 are the major fibrous collagens to form ECM in alveoli and type 4 majorly exists in the basal membrane for supporting epithelium [16, 17]. Enzymes, such as lysyl oxidase (LOX) and MMPs are involved in ECM post-translational modification. LOX families are copper-based amino-oxidases that help to crosslink fibrous ECM proteins such as collagens and elastin, which can increase the strength of ECM and prevent the degradation of fibrous components [18–20]. Increased expression of LOX is usually found in IPF patients compared to healthy control, as well as the small airway epithelium from smokers compared to non-smokers [21, 22].

Here, we hypothesized that sub-chronic exposure to ETS augments fibrogenesis induced by bleomycin (BLM) administration via collagen dynamic dysregulation. C57BL/6J and p16-3MR mice were exposed to ETS for 30 days and followed by bleomycin administration for 14 days. The duration of ETS exposure and post-bleomycin administration is based on previous studies to avoid emphysema development induced by CS exposure and recovery of scaring injury after 21 days post-bleomycin [13, 23–29]. To determine the age-related effects during ETS-induced fibrotic progression, young (4–5 months) and old (15–17 months) mice were used. Our study is novel/unique showing ETS and bleomycin induced via collagen-lysyl oxidase in age-dependent mechanism utilizing a reporter p16-3MR mechanism.

## Methods

### Animals and treatments

Young (4–5 months) and old (15–17 months) groups of *C57BL/6* and *p16-3MR* (male and female) mice were housed in a 12-hour-light/12-hour-dark cycle in the inhalation core facility at the University of Rochester. First, both young and old groups of each strain were exposed to ETS for a total of 30 days, 5 days/week, via the Teague machine with 100 total particular matter (TPM, mg/m^3^). Air-exposed control groups of mice were housed separately. After 6 weeks of exposure, bleomycin (BLM) (1.5U/kg) or PBS (50 µl) were oropharyngeal administered. Mice were sacrificed after 14 days. During the sacrifice, pentobarbital (100 mg/kg, intraperitoneal (i.p.) injection) was given, lung mechanics were measured during the sacrifice, and lung lobes were then snap-frozen for protein/RNA analysis. Lung lobes inflated with 1% low-melting agarose were used for histological analysis. Ganciclovir (GCV, 25 mg/kg) was given to *p16-3MR* mice via intraperitoneal (i.p.) injection of every alternative day for a total of 8 times post-BLM.

### Lung mechanics measurement

Lung mechanics (compliance, resistance, and elastance) were determined with the FlexiVent device (Scireq, Montreal, QC, Canada). Mice were first anesthetized with an intraperitoneal injection of sodium pentobarbital (100 mg/kg). After a tracheotomy, an 18-gauge cannula was introduced into the exposed trachea and attached to a computer-controlled rodent ventilator (FlexiVent; Scireq). Following the initial ventilation, compliance, resistance, and elastance were measured by a computer-generated program at 3cmH_2_O positive. The impedance of the equipment and tracheal tube was removed with appropriate calibration. These measurements were repeated 3 times for each animal [30].

### RNA isolation and NanoString measurement

Snap-frozen lungs were mechanically homogenized with QIAzol reagent, then each sample was mixed with chloroform and vortexed vigorously for 10 s. The samples were centrifuged at 20,000*g* for 15 min at 4 °C, after that, the aqueous phase was moved to new RNase-free tubes and mixed with isopropanol for 2 h at -20 °C. The mixtures were spun down at 20,000*g* for 15 min at 4 °C, and the supernatant was removed. RNA pellets were washed with 75% EtOH and then centrifuged at 20,000*g* for 15 min at 4 °C. Discarded the EtOH and re-suspended RNA with RNase-free water. All of the RNA samples were cleaned up by RNeasy Plus Mini Kit following the manufacturer’s protocol. RNA samples were kept at − 80 °C until analysis. RNA samples were quantified using a NanoDrop spectrophotometer (ND-1000, NanoDrop Technologies). A total of 100ng of RNA samples were used for NanoString analysis. We have used the nCounter Fibrosis Panel (Cat# XT-CSO-MFIB2-12, NanoString Technologies, Inc.) in this study. Briefly, the hybridization reaction mix was prepared by RNA samples, reporter codeset, and capture codeset. The mixtures were incubated at 65 °C for 16 h and stored at 4 °C until further profiling. NanoString running cartridge was used to load all the sample mixtures and performed gene expression profiling via nCounter SPRINT Profiler (NanoString Technologies, Inc.). After profiling, gene expression results were analyzed by nSolver 4.0 software, and reported as normalized count for data presentation and statistical analysis. Raw RCC files were uploaded to ROSALIND for advanced analysis and generated the signaling pathway scores as directed enrichment scores with at least 10% change, and *p* ≤ 0.05 were considered as significantly altered.

### Western blot

Snap-frozen lungs were mechanically homogenized in RIPA buffer (Cat# 89901 Thermo Fisher Scientific, USA) with protease inhibitor (Cat# 78442 Thermo Fisher Scientific, USA). Protein concentrations were quantified by Pierce BCA Protein Assay (Cat# 23225 Thermo Fisher Scientific, USA). Total 30 µg of protein was prepared for each lane for electrophoresis via precast gels (Cat# 5671085, Cat# 4568126 BioRad). After electrophoresis, proteins were transferred onto a nitrocellulose membrane (Cat# 1704159, BioRad) through a semi-dry system. The membranes were washed with tris-buffered saline containing 0.1% Tween 20 (TBS-T) for 15 min, then blocked with 5% bovine serum albumin (BSA) for 1 h at room temperature (RT). Next, the membranes were probed with primary antibodies overnight at 4 °C: anti-Collagen 1α1 (1:1000, NBP1-30054, Novus Biologicals); anti-Collagen 4α1 (1:1000, ab227616, Abcam); anti-LOX (1:1000 ab174316, Abcam); anti-fibronectin (1:1000, ab2413, Abcam); anti-MMP2 (1:1000, ab92536, Abcam); anti-C3 (1:1000, ab200999, Abcam), and the appropriate secondary antibody (Goat-Anti-Rabbit, 1:5000, Cat# 1706515, BioRad) for 1 h and 30 min at RT. After each antibody incubation, the membrane was washed 3 times in TBS-T at RT, 15 min per wash. The luminescence signals were developed using a chemiluminescence substrate (Cat# 34096, Thermo Fisher Scientific, USA). The membranes exposure and band intensities were detected via the Bio-Rad ChemiDoc MP imaging system (BioRad Laboratories, Hercules, CA, USA). Protein quantification was done by densitometry, and β-ACTIN (1:2500, ab20272, Abcam) was used as housekeeping control. Densitometry analysis was performed by Image Lab software (v4.1, BioRad, Hercules, California) and data was presented as fold change compared to the PBS group.

### H&E staining

 The H&E staining has been performed as previously described [31]. Briefly, lung sections (5 μm) were deparaffinized by xylene 3 times, 5 min each, and rehydrated with 100%, 95%, and 70% ethanol. Next, slides were quickly washed with water, and stained with hematoxylin for 1 min. Then sections were washed with water and then blued with 0.1% ammonia water for 10 s. Sections were washed with water, then soaked in Eosin for 1 min. Sections were washed with 95% ethanol for 1 min, then dehydrated with 95% and 100% ethanol, 2 times, 3 min each, and xylene, 3 times, 5 min each. Finally, slides were mounted in Permount mounting media (UN1294, Fisher Chemical) for microscopy.

### Gomori’s trichrome staining

Gomori’s trichrome staining has been used to quantify collagen deposition in the lungs from different experimental groups. Lung sections (5 μm) were deparaffinized, rehydrated, and finally rinsed with tap water. Sections were then subjected to Gomori’s trichrome staining (Thermo Fisher Scientific kit; Cat# 87020). In brief, sections were placed in Bouin’s solution at 56 °C for 1 h, and then washed with water until the yellow color is removed. Next, sections were immersed in Working Weigert’s Iron Hematoxylin Stain for 10 min and washed with water for 10 min. After, the sections were soaked in Trichrome Stain for 15 min and immediately after in acetic acid 1% for 1 min. The sections were rinsed with water for 30 s and dehydrated. Lastly, the slides were mounted by Permount (UN1294, Fisher Chemical) for microscopy observation. For each sample, 10 pictures (20x magnification) have been taken by Nikon microscopy (Eclipse Ni-U) and the amount of collagen has been quantified by Colour Deconvolution via FIJI ImageJ.

### Immunohistochemistry (IHC) staining

The staining protocol has been performed as previously described [13]. Lung sections (5 μm) were deparaffinized and rehydrated, and then the antigens were unmasked with antigen retrieval solution (10x) (S1699; Dako). Sections were blocked with 10% normal goat serum and then incubated overnight at 4 °C with primary antibodies: anti-Collagen 1α1 (1:100, NBP1-30054, Novus Biologicals); anti-Collagen 4α1 (1:100, ab227616, Abcam) and anti-LOX (1:50, ab174316, Abcam). Then, slides were soaked in 0.3% hydrogen peroxide for 15 min and washed with TBS. Goat anti-Rabbit Secondary antibody (1:1000, ab6721; Abcam) was applied to the section at RT for 1 h. Slides were developed with DAB Quanto Chromogen and Substrate (TA-125-QHDX; Thermo Fisher Scientific) and then counter-stained with hematoxylin. Finally, the slides were dehydrated and mounted for further analysis. Sections were obtained from a continuously sequential sectioning, and continued 3 sections were placed on the same glass slide. The sections were 5 μm away from each other. These markers have been evaluated in sequential sections and for each sample, 10 pictures (20x magnification) have been taken and compared between groups. Same areas from the lung sections stained with different markers were localized to understand the co-localization of the markers. The positive staining has been quantified using Colour Deconvolution via FIJI ImageJ.

### Statistical analysis

All the data were analyzed through one-way ANOVA with Šidák correction using GraphPad Prism software (version 9.0, GraphPad, San Diego, CA, USA). Results were presented as mean ± SEM, and *p* < 0.05 was considered significant.

### Study approval

The animal experiments performed in this study was following the standards from the United States Animal Welfare Act, NIH, and protocol approved by the Animal Research Committee of the University of Rochester.

## Results

### Bleomycin-induced lung injury and impaired lung mechanics were not affected by ETS exposure

**Fig. 1:**
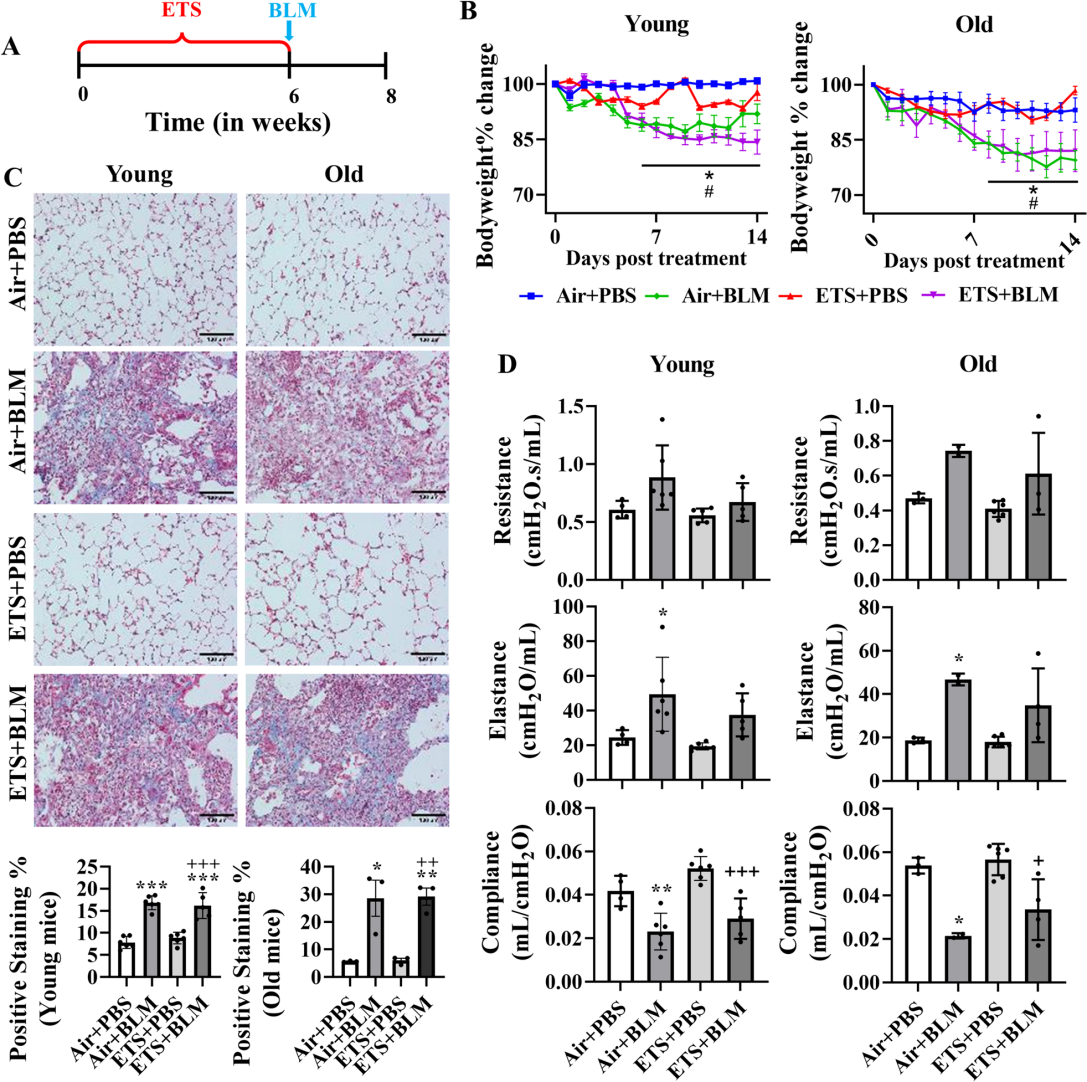
ETS exposure followed by bleomycin administration showed body weight decline, collagen deposition, and impaired lung function. **A** C57BL/6J mice (5 and 17 months old) were exposed to ETS for 30 days, and then administered with BLM, and all the parameters were analyzed 14 days-post BLM. **B** Body weight was monitored during 14 days post-BLM. C Lung mechanics (Resistance, Compliance, and Elastance) were measured during sacrifice on day 14 post-BLM. **D** Trichrome staining (20x, Scale bar = 100μm) was performed to calculate the collagen deposition (%). Data were presented as mean ± SEM (*n*=6/young group *n*=2-3/old group. *P<0.05, **P<0.01, ***P<0.001 compared with Air+PBS group; ^#^ *P*<0.05, ^# #^ *P*<0.01, ^# # #^ *P*<0.001 compared with Air+BLM group; ^+^ *P*<0.05, ^++^ *P*< 0.01, ^+++^ *P*<0.001 compared with ETS+PBS group)

Mice were exposed to ETS for 30 days and then BLM was administered oropharyngealy to evaluate how ETS exposure contributes to BLM-induced lung injury (Fig. 1A). Significant body weight declines were noticed after 7 days post-BLM injury in either young or old mice (Fig. 1B). ETS exposure only (ETS + PBS) did not show body weight alternation compared to the Air + PBS group, and ETS exposure did not affect the body weight reduction induced by BLM administration (ETS + BLM vs Air +BLM) in either young or old mice (Fig. 1B). H&E-stained lung sections showed similar lung injury between Air + BLM and ETS + BLM groups in both young and old mice. Limited airspace enlargement was seen in the ETS + PBS group compared to the Air + PBS group (Figure S1). There was no difference in BLM-induced lung injury between either young and old mice, or male or female mice (Figure S1). We also performed the Trichrome staining to determine the collagen deposition among different conditions. BLM (Air + BLM and ETS + BLM)-induced significant collagen deposition compared to Air + PBS or ETS + PBS groups, and ETS exposure did not affect the collagen deposition in the injured areas from either young or old mice (Fig. 1C). ETS exposure only (ETS + PBS) showed no altered collagen amount compared to the Air + PBS group in both young and old mice (Fig. 1C). The altered collagen deposition caused by BLM did not show either age or sex-based differences (Figure S2).

Lung mechanics (Resistance, Compliance, and Elastance) were measured on day 14 post-BLM administration. There was no significant alteration in resistance, while significantly decreased elastance and increased compliance was found in ETS + PBS compared to the Air + PBS group (Fig. 1D). BLM (Air + BLM) significantly increased the resistance and elastance and decreased compliance compared to the control group (Air + PBS). The decreased trend of resistance and elastance, and increased trend of compliance were found in the ETS + BLM group compared to Air + BLM group (Fig. 1D). Similar trends of lung mechanics were observed in both young and old mice (Fig. 1D).

### ETS exposure exacerbates bleomycin-induced abnormal collagen subtype biosynthesis and modification

**Fig. 2:**
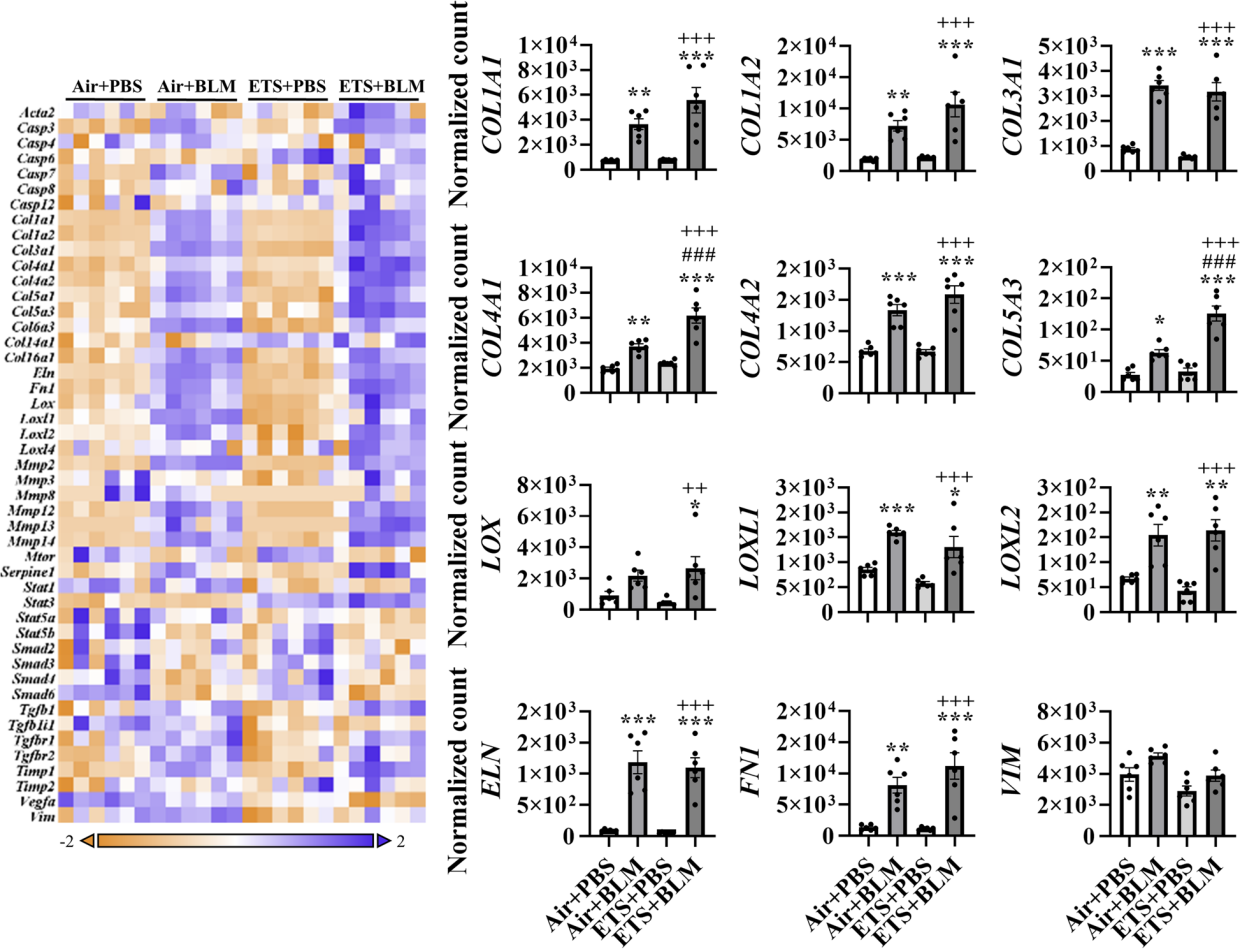
ETS exposure exaggerates collagen overexpression induced by bleomycin. Total RNA isolated from lung homogenates of young C57BL/6J mice (5 months old) was used to identify fibrotic gene transcript levels via NanoString nCounter Fibrosis panel. RNA expression levels were normalized by nSolver software, and the normalized count was used for statistical analysis. Dysregulated gene expression was shown as a heatmap focused on ECM protein and collagen dynamics. Selected gene expression is shown as bar graphs. Data are shown as mean ± SEM (*n*=6/group *P<0.05, **P<0.01, ***P<0.001 compared with Air+PBS group; ^#^ *P*<0.05, ^# #^ *P*<0.01, ^# # #^ *P*<0.001 compared with Air+BLM group; ^+^ *P*<0.05, ^++^ *P*< 0.01, ^+++^ *P*<0.001 compared with ETS+PBS group)

To further determine the role of ETS exposure in BLM-induced lung injury and dysregulated repair in young mice, RNA was isolated from lung homogenates, and fibrotic genes were measured via NanoString profiling (Fig. 2). BLM administration, either after ETS (ETS + BLM) or Air (Air + BLM) exposure, showed increased gene expressions of collagens. Sub-chronic ETS exposure followed by BLM administration augmented the upregulated transcription levels (ETS+BLM vs Air+BLM) of *COL1A1, COL1A2, COL4A1, COL4A2*, and *COL5A3.* Gene expression levels of *COL4A1* and *COL5A3* showed a significant increase in ETS + BLM compared to Air + BLM, while *COL1A1, COL1A2*, and *COL4A2* showed non-significant increase trends (Fig. 2). Other types of collagens, such as *COL3A1, COL5A1, COL14A1*, and *COL16A1*, were upregulated post-BLM injury with no significant difference after Air or ETS exposure. Other ECM genes, such as *ELN* and *FN1* showed upregulated gene expression levels after BLM, while no significant difference was noticed between ETS + BLM and Air + BLM groups. The transcription levels of lysyl oxidase and MMPs were also tested. Significantly increased gene expression levels of *LOX*, *LOXL1*, *LOXL2*, and *LOXL4* were found in Air + BLM and ETS + BLM compared to the Air + PBS group. There was no difference between Air + BLM and ETS + BLM groups (Fig. 2). A similar trend was noticed in gene expressions of MMPs *MMP2, MMP12, MMP13*, and *MMP14,* which were significantly upregulated in Air + BLM and ETS + BLM groups compared to the Air + PBS group, while no significant difference between Air + BLM and ETS + BLM (Figure S3). The MMPs inhibitor, *TIMP1*, showed increased gene expression level in Air + BLM and ETS + BLM groups compared to either Air + PBS or ETS + PBS groups (Fig. 2; Figure S3). One of the well-known fibrogenesis regulators, TGFβ signaling, was also tested in this study. Gene expression levels of *TGFB1*, *TGFB1I1*, *TGFBR1*, and *TGFBR2* were significantly increased post-BLM with either air (Air + BLM) or ETS (ETS + BLM) exposure compared to the respective PBS groups (Air + PBS and ETS + PBS) (Figure S3).

Based on the dysregulated gene list, pathway analysis was performed to determine how ETS affects the lung injury induced by BLM (Table 1). BLM administration showed significant upregulation in ECM degradation, collagen biosynthesis & modification, ECM synthesis, and PDGF signaling, all exacerbated by ETS exposure (Table 1). Besides, myofibroblast regulation and Senescence-Associated Secretory Phenotype (SASP) increased slightly after BLM administration, while ETS exposure with BLM exaggeratedly activated the signaling.

**Table 1:** Dysregulated pathways after ETS exposure and bleomycin administration

Term ID	Directed Enrichment Score		
	Air + PBS vs. ETS + PBS	Air + PBS vs. Air + BLM	Air + PBS vs. ETS + BLM
ECM Degradation	0.6191	3.3332	4.8184
Collagen Biosynthesis & Modification	-1.6072	3.0188	4.7557
ECM Synthesis	-1.5301	2.3663	3.7661
PDGF Signaling	-1.8479	1.364	2.3733
SASP	1.4557	0.8054	2.3648
Myofibroblast Regulation	-1.1383	0.3302	1.8717
Complement Activation	-0.3492	0.4872	1.621
Wnt	1.0543	-2.2748	-3.0824

**Fig. 3:**
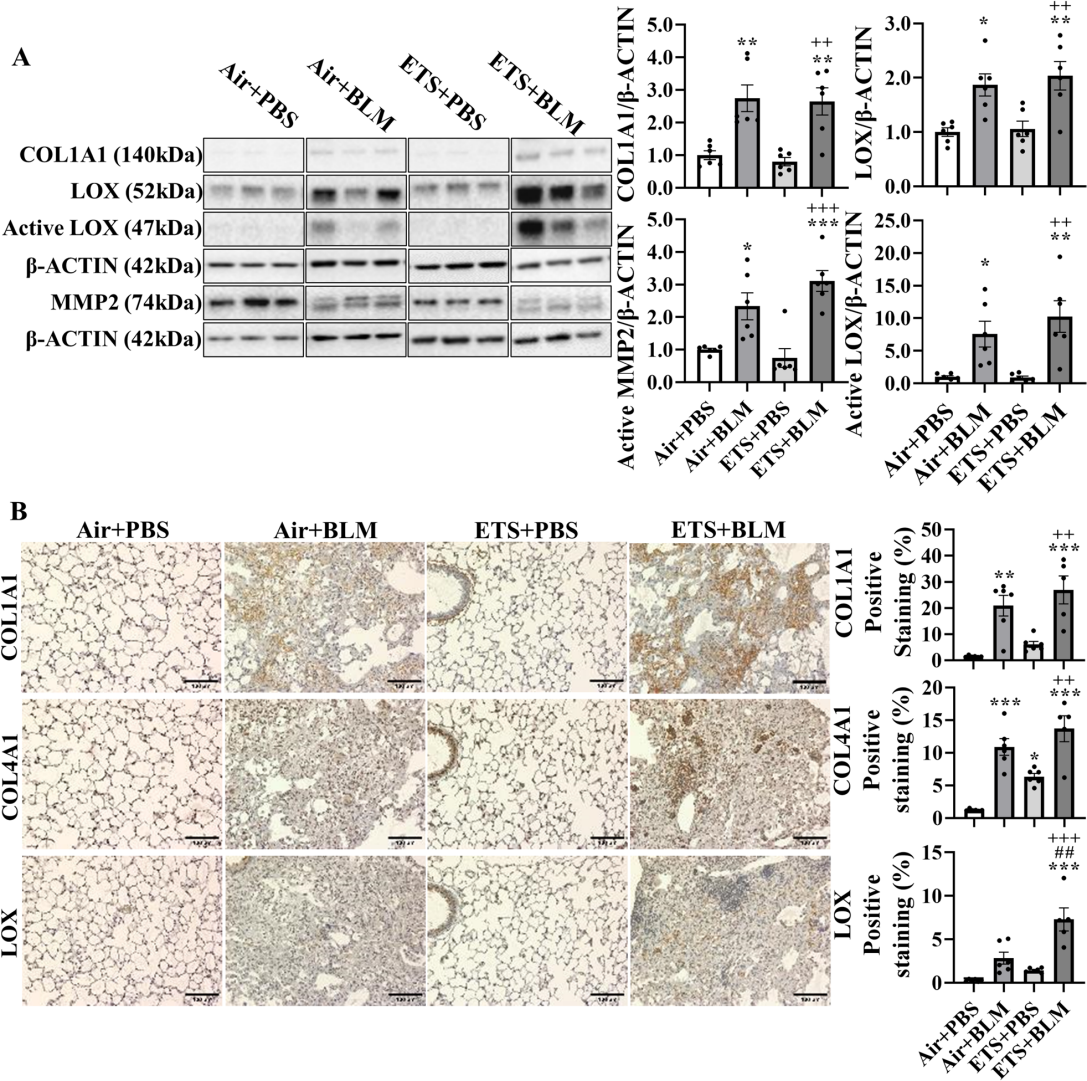
ETS exposure exacerbates bleomycin-induced overexpression of collagen and LOX. C57BL/6J mice (5 months old) were exposed to ETS for 30 days and then administered with BLM and these parameters were analyzed 14 days-post BLM. Protein expressions were analyzed by Western blotting from lung homogenates or by IHC staining on sequential sections (see methods). **A** Represented blot images (COL1A1, LOX, and MMP2) are shown. β-ACTIN was used as an endogenous control. Proteins expressions (fold change) are shown as bar graphs. Densitometry analyses are done individually. **B** Protein (COL1A1, COL4A1, and LOX) abundance and localization were quantified via IHC staining as a positive staining percentage. Data are shown as mean ± SEM (*n*=5-6/group *P<0.05, **P<0.01, ***P<0.001 compared with Air+PBS group; ^#^ *P*<0.05, ^# #^ *P*<0.01, ^# # #^ *P*<0.001 compared with Air+BLM group; ^+^ *P*<0.05, ^++^ *P*< 0.01, ^+++^ *P*<0.001 compared with ETS+PBS group)

Since the collagen biosynthesis and modification were activated by BLM administration and augmented in ETS exposure with BLM, protein expressions of COL1A1, COL4A1, LOX, and MMP2 were measured. Protein abundances of COL1A1, LOX, activated LOX, and activated MMP2 were upregulated after BLM administration, and ETS exposure showed no difference in total protein levels identified from immunoblot (Fig. 3A). Notably, the protein abundance of COL1A1 in males showed a significant increase in ETS + BLM compared to Air + BLM groups, while females showed a decreased trend (Figure S4). The significantly increased protein level of activated MMP2 was found in females after ETS exposure and BLM compared to Air + PBS, while no difference between Air + BLM and ETS + BLM groups was found in males (Figure S4). To determine the protein abundance in the lesion area, IHC staining was performed. The protein expressions of COL1A1, COL4A1, and LOX were increased after BLM administration following either air (Air + BLM) or ETS (ETS + BLM) exposure compared to Air + PBS or ETS + PBS respectively. The protein level of LOX in the lesion areas was significantly increased in the ETS + BLM group compared to Air + BLM, while COL4A1 and COL1A1 showed non-significant increased protein expression in the injury area of the ETS + BLM group compared to Air + BLM group (Fig. 3B).

### ETS exposure augmented cellular senescence induced by bleomycin treatment

**Fig. 4:**
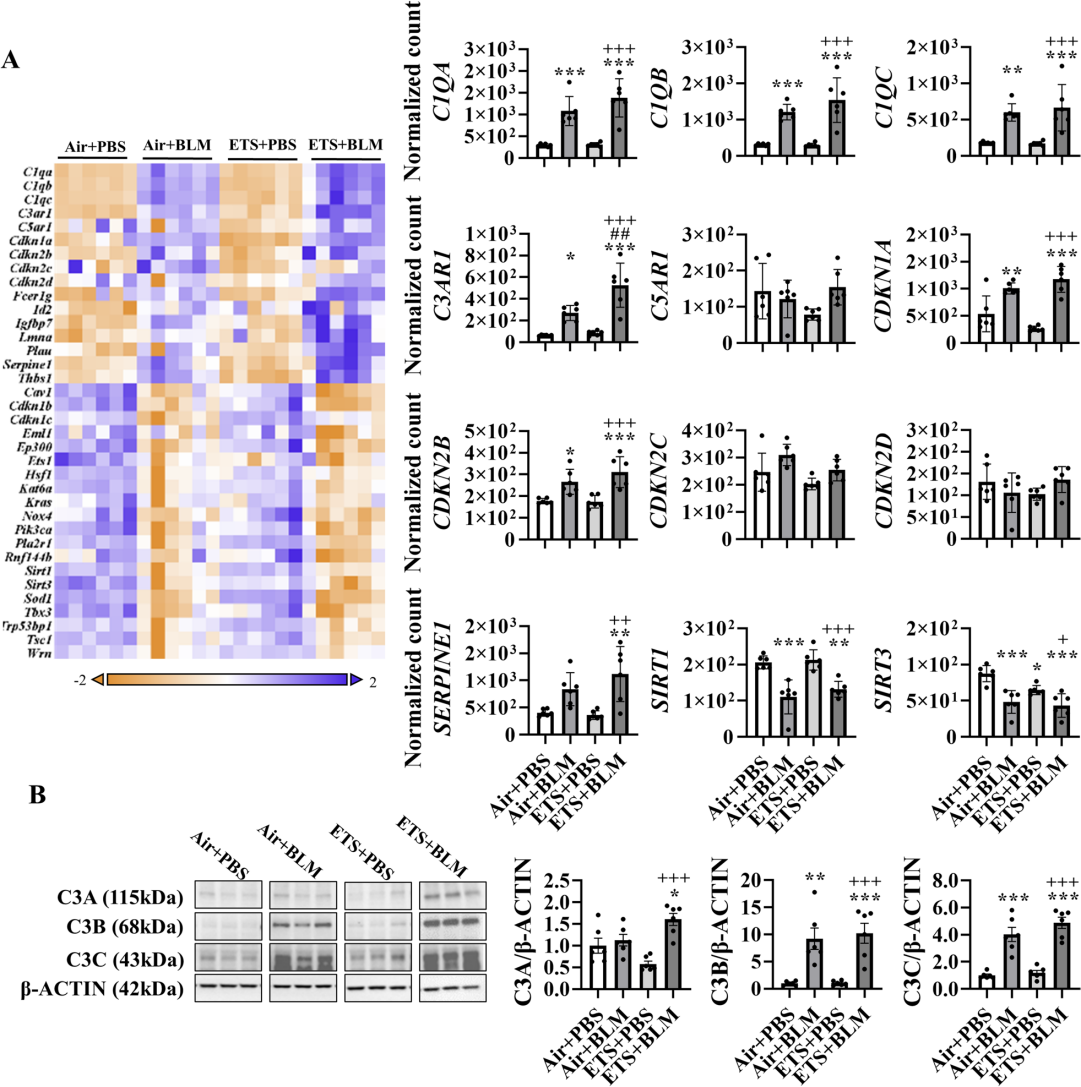
ETS exposure contributes to cellular senescence via activation of complement component C3.**A** Total RNA isolated from the lungs of young C57BL/6J mice (5 months old) was used to screen targets of interest. The heat map presents an overview of dysregulated targets, and the expression of selected genes is reported with bar graphs. **B** Protein was isolated from lung homogenates and C3 protein expression levels (C3A, C3B, and C3C) were analyzed by Western Blotting. β-ACTIN was used as endogenous control. data are shown as mean ± SEM (*n*=6/group. *P<0.05, **P<0.01, ***P<0.001 compared with Air+PBS group; ^##^ *P*<0.01 compared with Air+BLM group; ^+^ *P*<0.05, ^++^ *P*< 0.01, ^+++^ *P*<0.001 compared with ETS+PBS group)

Based on the signalling pathway analysis, senescence-associated secretory phenotype (SASP) was slightly activated after BLM administration (BLM + PBS), and ETS exposure followed by BLM (ETS + BLM) showed a further increase (Table 1). More importantly, ETS exposure only (ETS + PBS) showed a higher activation level than the BLM only group (Air + BLM) (Table 1). To further understand the dysregulated gene and protein expression levels, a customized NanoString panel focused on senescence genes was used to determine the dysregulated transcript levels. Gene expression levels of complement components (*C1QA*, *C1QB*, *C1QC*, *C3AR1*, and *C5AR1*) were upregulated after BLM administration either with air (Air + BLM) or ETS exposure (ETS + BLM) (Fig. 4A), while only *C3AR1* showed a significant difference between ETS + BLM and Air + BLM group. The gene levels of cyclin-dependent kinase inhibitors (*CDKN1A, CDKN2B, CDKN2C, CDKN2D, CDKN1B*, and *CDKN1C*) were tested as well. Increased *CDKN1A* and *CDKN2B* gene expression levels were found in Air + BLM and ETS + BLM groups compared to Air + PBS group, and ETS + BLM group showed further increased gene expression of *CDKN1A* when compared to Air + BLM group without significant difference (Fig. 4A). Other SASP markers, such as *SERPINE1*, showed significantly increased transcript level in ETS + BLM compared to either Air + PBS or ETS + PBS group, whereas the Air + BLM group showed an increased trend compared to Air + PBS without significance. The Sirtuin family genes, *SIRT1* and *SIRT3*, were also tested in this study, and gene expression levels were found significantly downregulated after BLM administration either with Air (Air + BLM) or ETS (ETS + BLM) exposure (Fig. 4A).

We found a significant difference in the gene expression of *C3AR1* between the ETS + BLM and Air + BLM groups. Then, we tested the protein abundance of C3 to confirm the activation of the complement system. A significant increase of C3 alpha chain (C3A) was seen after ETS exposure and BLM compared to Air + PBS or Air + BLM groups, and there is no change between Air + PBS and Air + BLM groups (Fig. 4B and Figure S5). A significantly increased protein level of C3A was identified in males, and no alternation of C3A protein abundance was identified in females (Figure S5). Increased protein abundances of C3 alpha chain fragment (C3B) and C3 alpha chain fragment 2 (C3C) were also significantly upregulated in Air + BLM and ETS + BLM groups compared to the Air + PBS group. ETS + BLM group showed further increased protein abundance of C3B and C3C compared to Air + BLM, but without significant difference (Fig. 4B), and similar trends were found in both males and females (Figure S5). Interestingly, the gene expression of *C3* showed a higher basal level in the old mice than in the young mice. However, a lower basal transcript level was detected in *C3AR1* gene expression (Figure S6). Despite the different basal levels of gene abundance of *C3* and *C3AR1*, the dysregulated trend under ETS exposure post-BLM showed similarly between young and old mice (Figure S6).

### ETS exposure does not affect bleomycin-induced fibrotic progression after chronologically aging

**Fig. 5:**
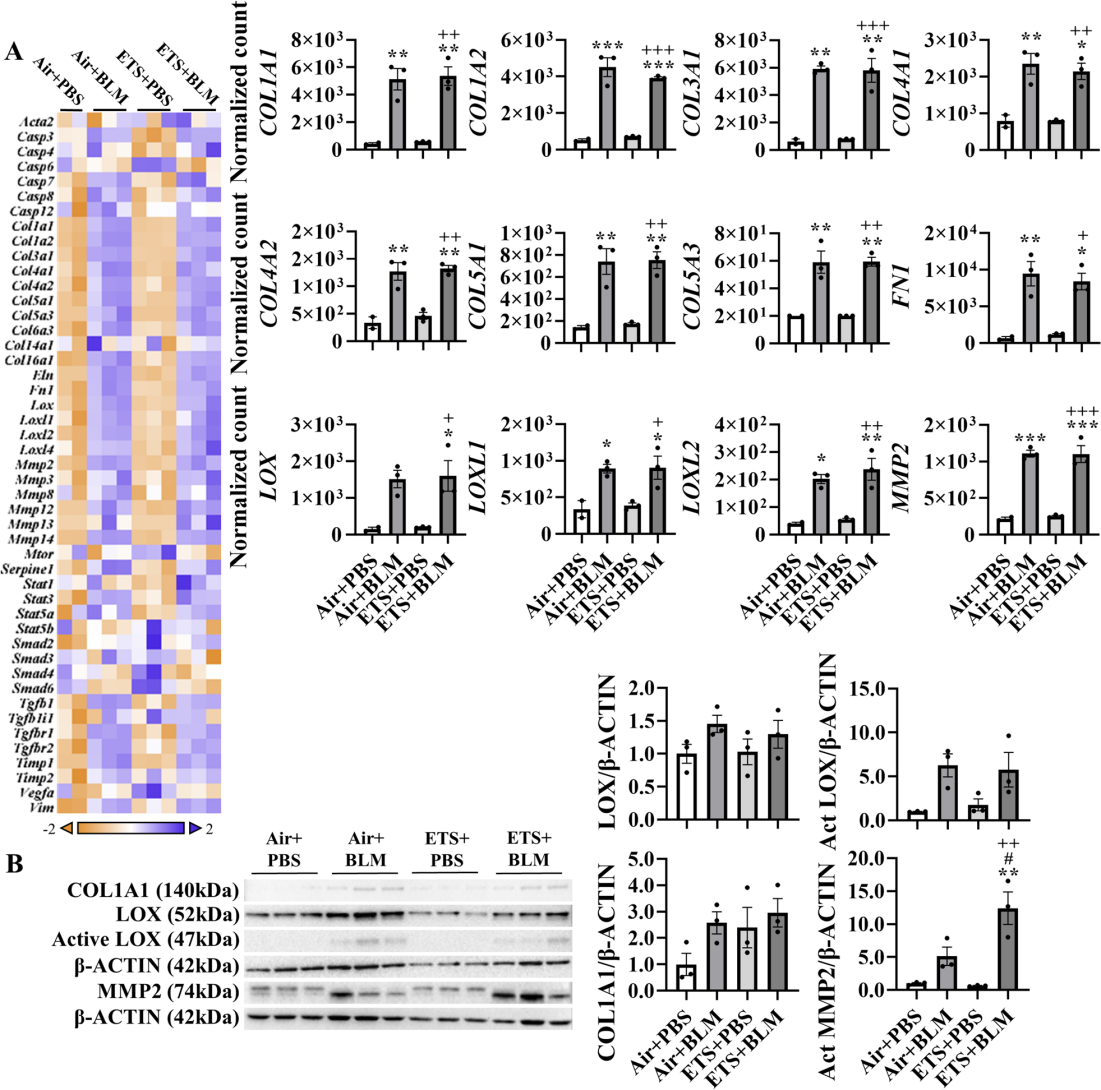
ETS exposure does not affect on bleomycin-induced fibrotic progression after chronological aging.Total RNA isolated from the lungs of old C57BL/6J mice (17 months old) was used to screen targets of interest. The heat map presents an overview of dysregulated targets (**A**), and the expression of selected genes is reported with bar graphs. Lung homogenates were probed with extracellular matrix (ECM) markers and protein expression was analyzed by Western Blotting. Represented blot images (COL1A1, LOX, and MMP-2) are shown (**B**). β-ACTIN was used as an endogenous control. Densitometry analyses are done individually, data are shown as mean ± SEM (*n*=2-3/group. *P<0.05, **P<0.01, ***P<0.001 compared with Air+PBS group; ^#^ *P*<0.05, ^##^ *P*<0.01, ^###^ *P*<0.001 compared with Air+BLM group; ^+^ *P*<0.05, ^++^ *P*< 0.01, ^+++^ *P*<0.001 compared with ETS+PBS group)

To determine the impact of ETS exposure on lung fibrotic progression after chronological aging, 18 months old mice were used in this study. Increased gene expression levels of collagens (*COL1A1*, *COL1A2*, *COL3A1*, *COL4A1*, *COL4A2*, *COL5A1*, and *COL5A3*) were observed after BLM administration, and ETS exposure did not affect the transcript levels (Fig. 4A). Similar to the gene levels of collagens, gene expressions of lysyl oxidase (*LOX*, *LOXL1*, and *LOXL2*) and *MMP2* showed upregulation in both Air + BLM and ETS + BLM groups compared to Air + PBS, whereas no alternation between Air + BLM and ETS + BLM groups (Fig. 5A). Other ECM genes, such as *FN1*, *ELN*, *SERPINE1*, and *VIM*, showed upregulated transcript levels after BLM administration and no alternation between Air + BLM and ETS + BLM groups (Fig. 5A). Similar to young mice treated with BLM, gene expression levels of MMPs (*MMP2*, *MMP3*, *MMP8*, *MMP12*, *MMP13*, and *MMP14*) were upregulated after BLM administration regardless followed by Air (Air + BLM) or ETS (ETS + BLM) exposure (Figure S7). In old mice, both *TIMP1* and *TIMP2* gene levels were upregulated after BLM administration, and there was no significant difference between Air + BLM and ETS + BLM groups (Figure S7). The gene levels of TGFβ and its receptors (*TGFB1*, *TGFB1I1*, *TGFBR1*, and *TGFBR2*) showed upregulation in Air + BLM and ETS + BLM group compared to either Air + PBS or ETS + PBS groups, and there was no significant difference between Air + BLM and ETS + BLM groups (Figure S7).

Besides the gene expression measurement, protein abundances were also tested from lung homogenates (Fig. 5B). The increased protein level of COL1A1 was found in Air + BLM, ETS + PBS, and ETS + BLM groups compared to the Air + PBS group, while there was no significant difference among the groups (Fig. 5B). Protein abundance of LOX and activated LOX were increased after BLM administration, and ETS exposure did not alter the BLM-induced protein levels upregulation (Fig. 5B). Increased protein abundance of activated MMP2 was found after BLM and ETS exposure followed by BLM (ETS + BLM) augmented the activated MMP2 protein level compared to the Air + BLM group (Fig. 5B). When normalizing the gene expression levels from young and old mice, similar trends of differential gene expression after ETS exposure post- BLM for all different subtypes of collagens (Figure S8**-** S9). Most of the collagens gene dysregulation trends are similar between young and old mice under different conditions, while transcript levels of *COL3A1*, *COL5A1*, and *COL14A1* showed further upregulation post-BLM in old mice compared to young mice. However, the basal gene expression of *COL4A1* showed lower in old mice despite the dysregulation trends being similar between young and old mice (Figure S8). Unlike the gene expression of collagens, the transcript levels of lysyl oxidases and proteases showed decreased basal levels in older mice, although the dysregulation trend is comparable between young and old mice (Figure S8**-** S9).

### Removing of p16 positive cells did not recover bleomycin-induced bodyweight decline and impaired lung function

**Fig. 6:**
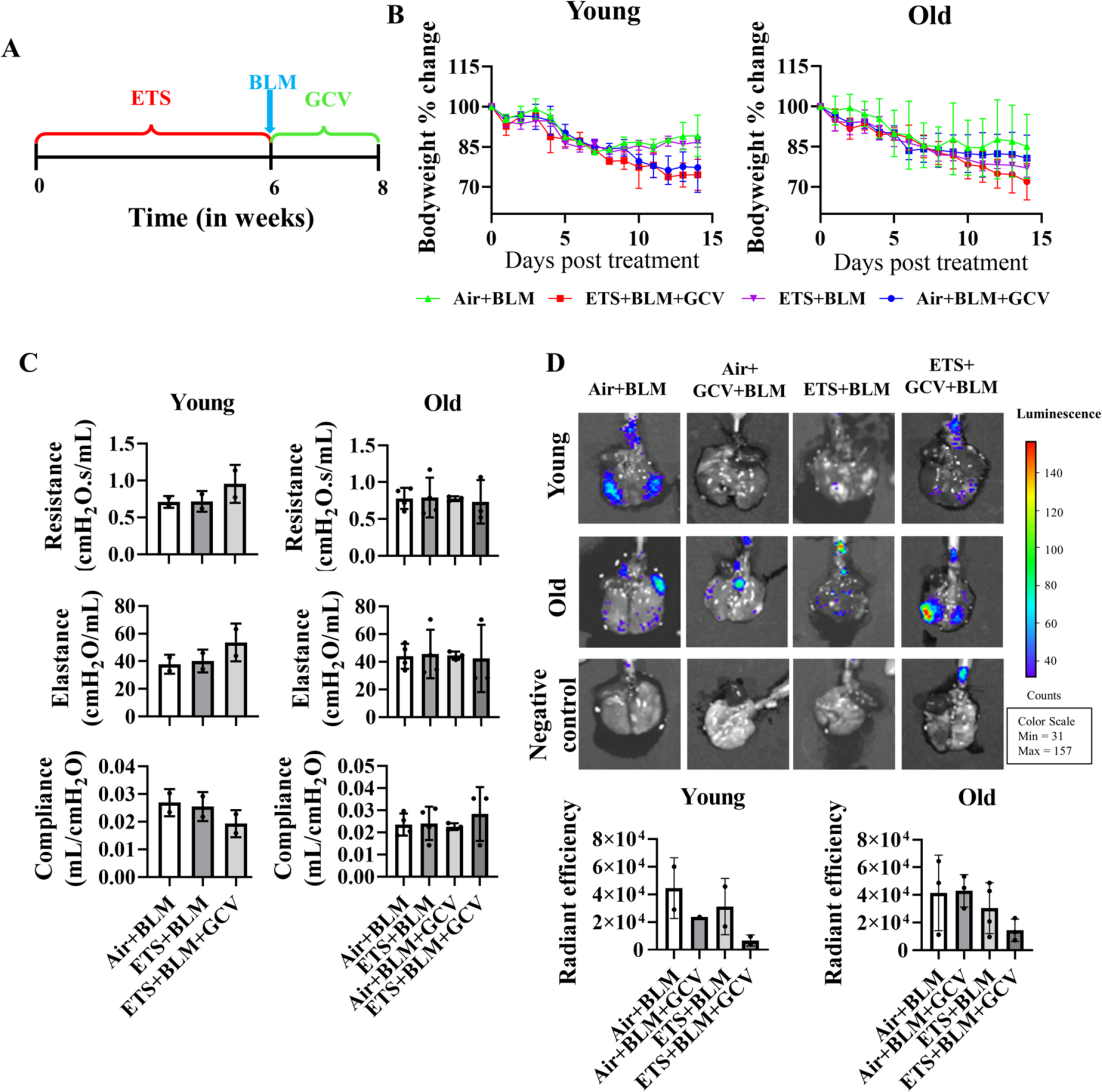
Removing of p16-positive cells did not recover bleomycin-induced body weight decline and impaired lung function.p16-3MR mice (5 months and 17 months) exposed to ETS for 30 days, administered with BLM and GCV for 14 days. **A** Illustration of animal treatment in this study. **B** Body weight was monitored during 14 days post-BLM (**C**) Lung mechanics (Resistance, Compliance, and Elastance) were measured during the sacrifice at day 14 post-BLM. **D** Radiant efficiency was measured via IVIS during sacrifice. Data were presented as mean ± SEM (*n*=2/young group *n*=3-4/old group)

Since the ETS exposure augmented premature senescence induced by BLM, we also utilized p16-3MR mice, which can remove the p16 overexpressing senescent cells with the help of ganciclovir (GCV). GCV was given to individual mice during BLM administration after ETS exposure on alternative days (Fig. 6A). After dosing GCV, there is no difference in body weight decline in either young or old mice, and there is no difference in lung mechanics (resistance, compliance, and elastance) among different experimental groups in either young or old mice (Fig. 6B-C). Administration of GCV showed non-significantly decreased p16 expressed cells in Air + BLM + GCV and ETS + BLM + GCV groups compared to Air + BLM and ETS + BLM groups, respectively, in young mice (Fig. 6D). However, there was no difference of p16 expressed cells in Air + BLM and Air + BLM + GCV groups in old mice, and a decreased trend was found in ETS + BLM + GCV compared to ETS + BLM groups, without significance (Fig. 6D).

## Discussion

Exposure to ETS, a mixture of tobacco smoke from the side-stream of cigarettes and the exhaled smoke from active smokers, has shown correlations with asthma and COPD development. However, no clinical evidence showed how ETS exposure contributes to fibrogenesis development [12, 32]. Recent animal studies have described that CS exacerbates the fibrotic progression induced by different causes such as bleomycin, lipopolysaccharides (LPS), polyhexamethylene guanidine, and influenza A virus (IAV) [3, 6, 7, 33, 34]. CS exposure followed by bleomycin administration showed significantly increased hydroxyproline and collagen deposition compared to bleomycin alone [3, 6]. CS exposure also augments acute inflammatory responses induced by LPS treatment to fibrotic lesion formation, with increased levels of TGFβ, αSMA, and collagens [34]. Here, we found that ETS exposure exacerbates collagen overexpression and activation of C3 complement signaling induced by bleomycin in relatively younger ages. After chronologically aging, ETS exposure showed no effects on bleomycin-induced collagen content upregulation. Our results showed that ETS exposure could exaggerate collagen biosynthesis and modification through MMPs and LOX.

Tobacco smoke is a well-known risk factor for COPD/emphysema development, usually with decreased elastance, increased compliance, and airspace enlargement. Previously, we have shown that main-stream CS exposure induced increased compliance, mean linear intercept (Lm), and inflammatory cell-flux infiltration [13]. In this study, we have noticed slightly increased compliance, decreased elastance after ETS exposure without significance, and limited airspace enlargement compared to air control. Since ETS is considered a low dose of tobacco smoke, a longer exposure duration and more robust tobacco-smoke exposure might be needed to develop emphysema. After bleomycin administration, increased elastance and decreased compliance were observed, which are the hallmarks of fibrotic progression. Interestingly, slightly decreased elastance and increased compliance were noticed in the ETS + BLM group compared to Air + BLM, which might be the combined effects of tobacco smoke exposure and bleomycin, which showed as a phenomenon of combined pulmonary fibrosis and emphysema (CPFE). It has been confirmed clinically that patients with CPFE have an intermediated value of lung mechanics parameters between patients with emphysema and fibrosis [35]. A similar trend of dysregulated lung mechanics was found in both young and old, which showed that ETS exposure and bleomycin administration-induced lung injury is age-independent. We also found increased collagen deposition in the lung lesion areas caused by bleomycin, while no overall collagen deposition was promoted after ETS exposure, which does not concur with the previous study [6]. Subtypes of collagen: *COL4A1* and *COL5A1* showed significantly increased RNA expression levels in ETS + BLM compared to the Air + ETS group, and non-significant increased *COL1A1*, *COL1A2*, and *COL4A2* were found as well in the ETS + BLM group compared to Air + BLM. Besides, our study also showed further increased protein level of COL1A1 in males rather than females in the ETS + BLM group compared to the Air + BLM group, which partially agreed that males were more vulnerable during fibrogenesis [36]. A recent study showed that CS exposure aggravated the collagen expression induced by IAV infection [7]. The same study has shown CS exposure exaggerated the IAV infection-activated fibroblast to myofibroblast differentiation with increased TGFβ and αSMA [7]. Another study also showed CS exposure mediated fibrotic progression and exacerbation might occur through TGFβ/Smad2 signaling pathway [6]. Our study has further confirmed that collagen dynamic was also one of the possible pathways contributing to fibrogenesis exacerbation due to ETS exposure. Although we noticed increased gene levels of TGFβ and its receptors, no significant difference was identified between Air + BLM and ETS + BLM groups. More studies are required to understand how tobacco smoke exposure contributes to fibrogenesis development.

Our gene expression results and pathway analysis showed further activation of ECM degradation, collagen biosynthesis and modification, and ECM synthesis. We, therefore, focused on MMPs and lysyl oxidases, which are the enzymes responsible for ECM remodeling. Increased protein abundance of MMP2 was found either in lung fibrosis animal models or fibrotic clinical samples [37]. Our results showed increased gene and protein levels of MMP2 which agreed with the previous study, and our results further illuminated that ETS exposure further exaggerated active MMP2 protein levels in females, and no significant upregulation in Air + BLM compared to the Air + PBS group. However, the upregulation of MMP2 was found in males treated with bleomycin regardless of ETS or Air exposure. It has been well-stated that female has better survival during fibrogenesis than male, and our results contribute to this statement that upregulated MMP2 during fibrogenesis is only found in males. ETS exposure could be one of the factors in helping develop fibrotic progression in females [36].

Another enzyme tested in this study is lysyl oxidase, a copper-dependent amine oxidase that helps to stabilize the ECM structure via crosslinking collagen and elastin fibers [38]. Upregulation of lysyl oxidase has been identified from the fibrotic lesion in lung fibrosis patients and in bleomycin-induced lung fibrotic injury [39]. Upregulated lysyl oxidases during fibrogenesis drive severe crosslinking of collagen and elastin fibers, preventing degradation of fibrous matrix and contributing to irreversible scarring [40]. In this study, we identified that fibrogenesis exacerbation induced by ETS exposure could be through lysyl oxidase-mediated abnormal collagen dynamics. Although the gene expression levels of *LOX*, *LOXL1*, and *LOXL2* showed no significant difference between the ETS + BLM and Air + BLM groups, a significantly increased protein abundance of LOX was found especially in the fibrotic lesion area in the ETS + BLM group compared to Air + BLM group. More importantly, ETS exposure impacts bleomycin-induced upregulated protein abundance of active LOX in males only, whereas no difference is identified in females. Our results of lysyl oxidase differentiated expression in different groups in a sex-dependent manner contribute to the statement that males showed worse lung injury during fibrotic progress than females [36]. The inflammation responses during fibrogenesis showed higher in males than females with exacerbated neutrophil and eosinophil infiltration and aggravated inflammatory cytokine levels [41]. The increased inflammation baseline could be one of the causes of higher susceptibility and worse lung injury in males. Our previous studies showed a sex difference in ECM remodeling following lung inflammation [42, 43]. One of the potential explanations for sex differences in fibrotic progression might be the male sex hormone. Androgen has been proven to exacerbate the airway inflammation induced by LPS [44], and removing the androgen in male mice attenuated the fibrotic responses induced by bleomycin [45].

Other than sex-dependent manner, we also studied whether aging affects ETS exposure on bleomycin-induced fibrogenesis. Our results show no significant alternation of most of the fibrotic markers except for active MMP2. Our results showed that ETS exposure has no significant impact on the exacerbation of fibrotic progression, while ETS exposure augments collagen synthesis and modification during bleomycin-induced fibrogenesis in relatively younger mice. All collagens are not pro-fibrotic as type IV collagen is a normal part of basement membranes including the alveolar basement membrane, which our study delineates.

Since COPD and IPF are both senescence/aging-related diseases [46, 47], we have customized a senescence-focused NanoString transcriptomic panel to understand the effect of ETS exposure and bleomycin-induced cellular senescence. During cellular senescence, molecular checkpoint systems responsible for DNA damage, cell cycle, apoptosis, and other routine cellular functions are disrupted and finally become irreversible [47, 48]. After bleomycin administration, our results showed increased gene expressions of different cyclin-dependent kinase inhibitors (*CDKN1A, CDKN2B*, and *CDKN2C*). A non-significant further increased *CDKN1A* was found in ETS + BLM compared to the Air + BLM group. The protein encoded by *CDKN1A* is p21, which can cause cell cycle arrest by inhibiting CDK2 and CDK4 [49]. Either CS exposure or bleomycin alone has been reported to increase *CDKN1A* in the alveolar epithelium [15]. Our result agreed with the previous study and further showed that ETS exposure could exacerbate bleomycin-induced *CDKN1A* upregulation. Another SASP gene, *SERPINE1*, showed upregulation after bleomycin administration and a further upward trend was found in the ETS + BLM group compared to the Air + BLM group. It has shown that the upregulation of *SERPINE1* induced the phosphorylation of p53 and increased the protein level of p21, which activated p53-p21-Rb-associated cell cycle repression [50]. Our results confirmed that exacerbated upregulation of *SERPINE1* by ETS could augment the senescence process induced by bleomycin.

Besides the CDKN family and SERPINE1, the transcriptomic panel also showed irregular activation of the complement system, which plays an important role during cellular senescence and aging [51]. It has been shown that complement C3 level is positively correlated with age, and inhibition of C3 could prevent senescence progression in the renal system [52]. Multiple studies focus on complement system development in emphysema and IPF [53, 54]. Removing C3 or C3A receptor (C3AR) helped prevent inflammation and airspace enlargement caused by chronic CS exposure [53]. In the same study, increased protein levels of C3 and C3AR were increased after chronic CS exposure [53]. However, our results showed no change in the protein level of C3A and gene expression of *C3AR* after ETS exposure. Longer exposure duration and higher doses of ETS should be considered to activate C3 and C3AR. Another study showed that an increased protein abundance of C3A was found after bleomycin treatment [54]. Increased protein abundance of C3A and C5A protein drives the expression of FN1 and αSMA in lung fibroblast, which are the hallmarks of fibrogenesis [54]. In the same study, blocking C3AR attenuated lung injury and upregulated collagens induced by bleomycin treatment [54]. Our results agreed with the increased expression of C3 and C3AR, and we further identified that ETS exposure exacerbates the bleomycin-induced activation of C3AR. Besides, the protein abundance of C3A showed a significant increased and non-significant increased trend for C3B and C3C in ETS + BLM compared to Air + BLM group in male mice, whereas females either showed no difference or decreased trend. Our results in complement components again confirmed that males were more vulnerable during fibrogenesis than females. However, limited SASP markers were dysregulated significantly, adjusting the duration post-BLM might help emphasize the exacerbation of SASP by ETS exposure.

As mentioned before, fibrogenesis is an aging disease usually occurring in elders. However, based on our normalized gene expressions of collagen, lysyl oxidase, and proteases, older mice did not show significant dysregulation levels than younger mice in most fibrotic markers. It has been reported that the bleomycin-induced fibrosis model serves better in the old mice, in which the chronological aging could delay fibroblast-dominated repair capacity, hence inducing persistent scarring and ECM deposition, whereas young mice showed recovery in lung scarring [55, 56]. Bleomycin treatment for 14 days might not be enough for mimicking the fibrogenesis progression in humans since it is a chronic lung disease. A longer duration post-BLM (28 days) in old mice could help produce persistent scar tissue in the lungs, which is the pathological phenomenon of lung fibrogenesis. Besides, removing ETS-induced overexpression of p16, one of the senescence markers, did not help resolve the lung injury induced by bleomycin, indicating that targeting p16 might be efficient for emphysema development but not fibrotic progression [57]. However, the number of aged mice used in this study was limited, resulting in an insufficient description of how ETS exposure affected the progression of bleomycin-induced lung fibrosis. Lacking of enough samples here also limited the power to demonstrate the sex difference in aged mice during the fibrogenesis exacerbation.

In conclusion, we showed that ETS exposure exacerbated bleomycin-induced overexpression of collagen subtypes, lysyl oxidase, and C3A-receptor signaling. Removing p16 high-expressed cells did not help alleviate the lung injury induced by bleomycin. More importantly, our results showed that male mice were more susceptible than females during fibrogenesis exacerbation. Our study provided potential signaling that ETS exposure not only activated the TGFβ/SMAD2 pathway but also C3A-receptor signaling, which might be the potential reason for exacerbated abnormal collagens dynamics during fibrogenesis.

### Supplementary Information

**Supplementary Material 1.**

## Data Availability

Data are available upon request from the corresponding author.
