# Supplementary material for: Environmental tobacco smoke exposure exaggerates bleomycin-induced collagen overexpression during pulmonary fibrogenesis
Source: J Inflamm (Lond). 2024 Mar 20;21:9. doi: 10.1186/s12950-024-00377-y (PMC10956237; doi:10.1186/s12950-024-00377-y)
Supplement: Supplementary file 1 — Supplementary Material 1. [file 12950_2024_377_MOESM1_ESM.pdf]

Supplementary information

Environmental tobacco smoke exposure exaggerates bleomycin-induced collagen overexpression during pulmonary fibrogenesis

[1]Qixin Wang[$], [1]Chiara Goracci[$], [2]Isaac Kirubakaran Sundar, [1]Irfan Rahman

[1]Department of Environmental Medicine, University of Rochester Medical Center, Rochester, NY, USA.

[2]Department of Internal Medicine, Division of Pulmonary, Critical Care and Sleep Medicine, University of Kansas Medical Center, Kansas City, KS, USA.

[$]These authors contributed equally

Address for Correspondence:
* Irfan Rahman, Ph.D.
Department of Environmental Medicine
University of Rochester Medical Center
Box 850, 601 Elmwood Avenue
Rochester 14642, NY, USA
E-mail: irfan_rahman@urmc.rochester.edu

Supplementary figures and legends

Figure S1

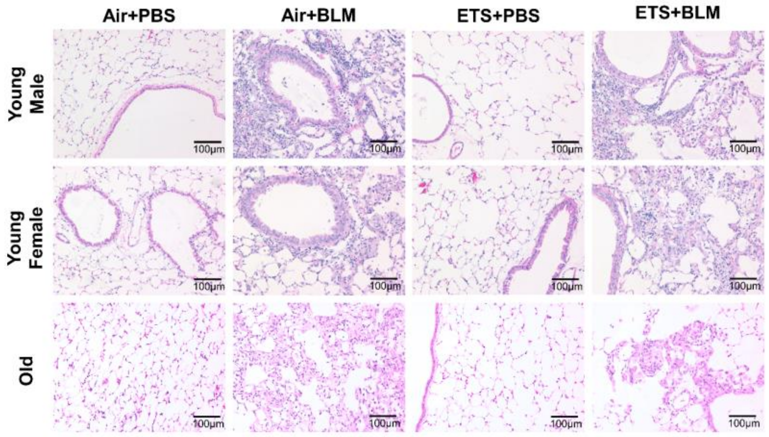

Figure S1: Lung injury induced by ETS exposure and bleomycin administration did not show sex- or age-based differences

Lung sections were prepared and performed for H&E staining to observe the lung injury induced by ETS exposure and Bleomycin treatment (20x, Scale bar = 100μm).

Figure S2

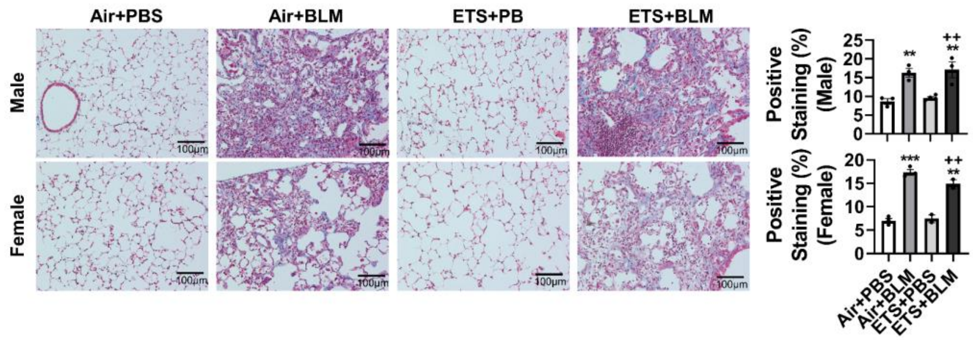

Figure S2: ETS exposure did not exaggerate bleomycin-induced collagen deposition in either male or female

Lung sections were prepared and performed for Trichrome staining to observe the collagen deposits induced by ETS exposure and Bleomycin treatment (20x, Scale bar = 100μm). The positive staining area percentage was calculated via ImageJ. Male and female mice data were separated. Data are shown as mean ± SEM. (n=2-4, **P<0.01, ***P<0.001 compared with Air+PBS group; ++P< 0.01 compared with ETS+PBS group).

Figure S3

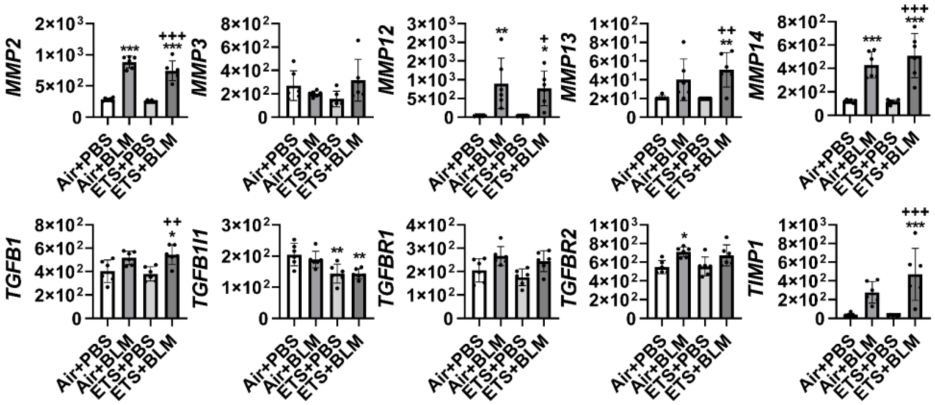

Figure S3: Dysregulated fibrotic genes after ETS exposure and bleomycin administration

RNA was isolated from lung homogenates and used to measure the fibrotic gene expression levels via NanoString panel (nCount Fibrosis v2) via nCounter SPRINT profiler. Normalized RNA counts were generated via nSolver 4.0, and normalized counts were used for data representation and statistical analysis. Data are shown as mean ± SEM. (n=6, *P<0.05, **P<0.01, ***P<0.001 compared with Air+PBS group; $^{+}$P<0.05, $^{++}$P< 0.01, $^{+++}$P<0.001 compared with ETS+PBS group).

Figure S4

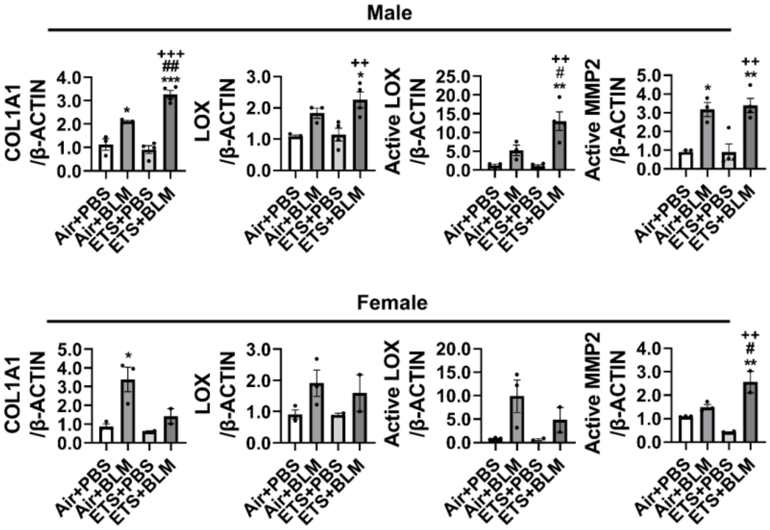

Figure S4: ETS exposure augments protein expression of COL1A1 and active LOX up-regulation induced by bleomycin in male

Protein was isolated from lung homogenates and used to measure the protein abundance of COL1A1, LOX, active LOX, and active MMP2 via immunoblot. Male and female mice were separated to distinguish the sex-based differences. β-ACTIN was used as an endogenous control. Densitometry analyses are done individually, data are shown as mean ± SEM (n=2-4, *P<0.05, **P<0.01, ***P<0.001 compared with Air+PBS group; $^{\#\#}$P<0.01, compared with Air+BLM group; $^{+}$P<0.05, $^{++}$P< 0.01, $^{+++}$P<0.001 compared with ETS+PBS group).

Figure S5

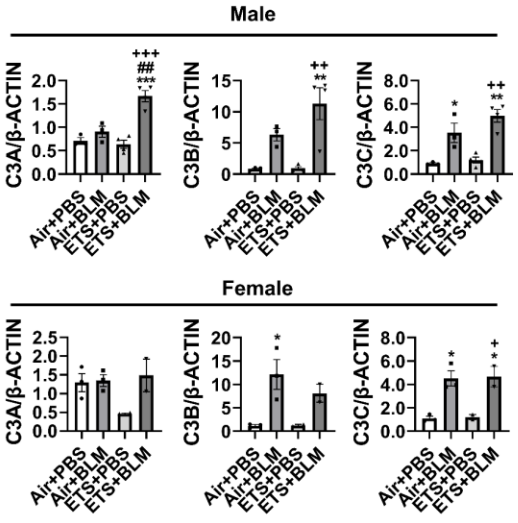

Figure S5: ETS exposure augments bleomycin-induced upregulation of protein expression of C3a in male

Protein was isolated from lung homogenates and used to measure the protein abundance of C3A, C3B, and C3C via immunoblot. Male and female mice were separated to determine the sex-based differences. β-ACTIN was used as an endogenous control. Densitometry analyses are done individually, data are shown as mean ± SEM (n=2-4, $^{*}P<0.05$, $^{**}P<0.01$, $^{***}P<0.001$ compare with Air+PBS group; $^{\#\#}P<0.01$, compared with Air+BLM group; $^{+}P<0.05$, $^{++}P< 0.01$, $^{+++}P<0.001$ compared with ETS+PBS group).

Figure S6

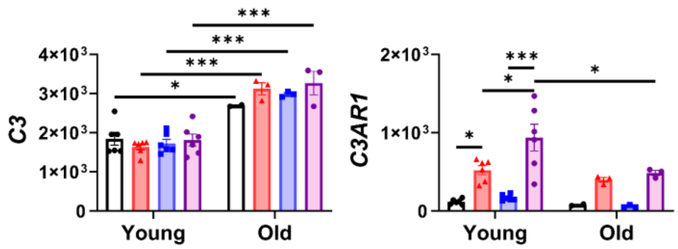

Figure S6: Differential gene expression of C3 and C3AR1 in young and old mice after ETS exposure and bleomycin treatment

RNA isolated from lungs was used to quantify the expression of complement genes via NanoString panel (nCount Fibrosis v2) and nCounter SPRINT profiler. Normalized RNA counts were generated, and both RNA expressions from young and old mice were normalized together in nSolver 4.0. Data are shown as mean ± SEM. (n=2-6, *P<0.05, **P<0.01, ***P<0.001).

<u>Figure S7</u>

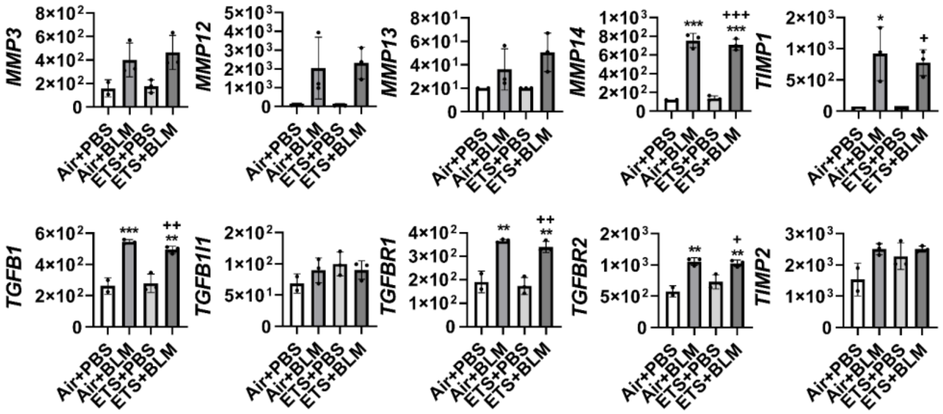

Figure S7: Dysregulated fibrotic genes after ETS exposure and bleomycin administration in chronologically aged mice

RNA was isolated from lung homogenates from 17 month-old mice and used to measure the fibrotic gene expression levels via NanoString panel (nCount Fibrosis v2) via nCounter SPRINT profiler. Normalized RNA counts were generated via nSolver 4.0, and normalized counts were used for data representention and statistical analysis. Data are shown as mean ± SEM. (n=2-3, **P<0.01, ***P<0.001 compared with Air+PBS group; $^{+}$P<0.05, $^{++}$P< 0.01, $^{+++}$P<0.001 compared with ETS+PBS group).

Figure S8

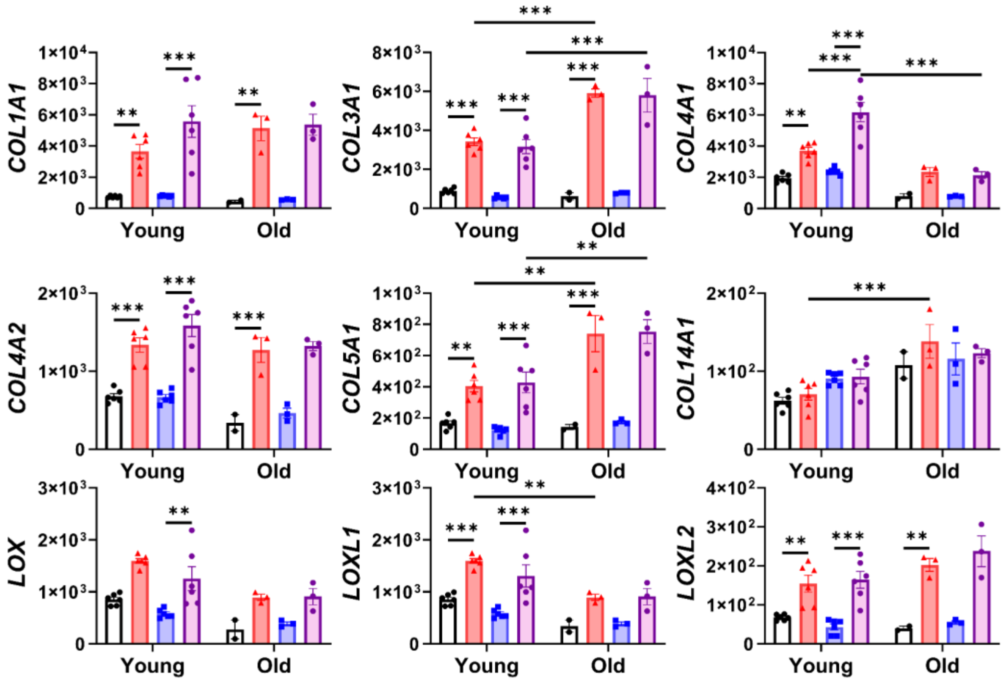

Figure S8: Differential gene expression of collagens and lysyl oxidases in young and old mice after ETS exposure and bleomycin treatment

RNA isolated from lungs was used to quantify the expression of collagens and lysyl oxidase genes via NanoString panel (nCount Fibrosis v2) and nCounter SPRINT profiler. Normalized RNA counts were generated, and both RNA expressions from young and old mice were normalized together in nSolver 4.0. Data are shown as mean ± SEM. (n=2-6, *P<0.05, **P<0.01, ***P<0.001).

Figure S9

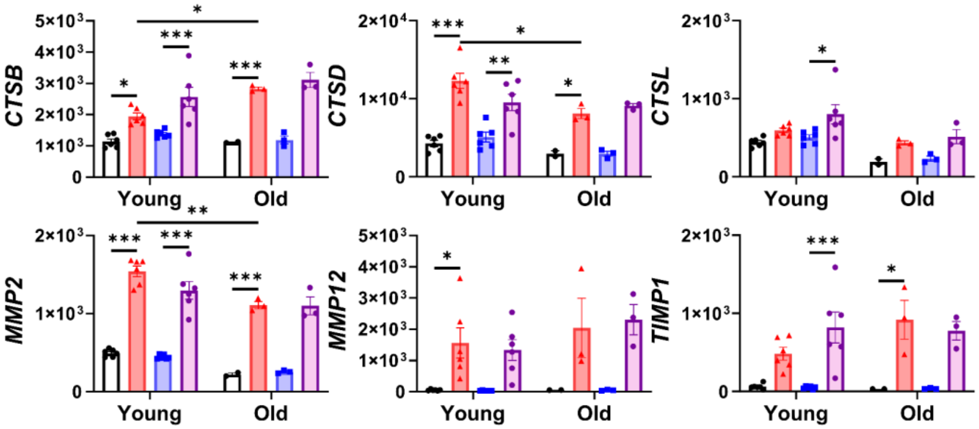

Figure S9: Differential gene expression of proteases and protease inhibitors in young and old mice after ETS exposure and bleomycin treatment

RNA isolated from lungs was used to quantify the expression of senescence genes via NanoString panel (nCount Fibrosis v2) and nCounter SPRINT profiler. Normalized RNA counts were generated, and both RNA expressions from young and old mice were normalized together in nSolver 4.0. Data are shown as mean ± SEM. (n=2-6, *P<0.05, **P<0.01, ***P<0.001).
